# Double bad luck: pulmonary embolism and vaginal bleeding - a case report

**DOI:** 10.1186/s12245-024-00639-9

**Published:** 2024-05-13

**Authors:** Sarah Shiba, Jan Van Keer

**Affiliations:** 1 Department of Anesthesia, Erasmus Hospital, Rte de Lennik 808, Brussels, 1070 Belgium; 2 Department of Cardiology, AZ Turnhout, AZ Turnhout, Rubensstraat 166, Turnhout, 2300 Belgium

## Abstract

**Background:**

Pulmonary embolism is a common and potentially fatal condition. Exogenous estrogens in contraceptives are associated with an increased risk of venous thrombo-embolism. However, discontinuation of a combined oral contraceptive can lead to severe withdrawal bleeding in an anticoagulated patient.

**Case presentation:**

We report a case of a 47-year-old female who presented to the emergency room with a two-day history of worsening shortness of breath and chest pain. Her chronic medication included a combined oral contraceptive pill. Transthoracic echocardiogram showed pulmonary hypertension and right ventricular dilatation. Computerized tomography scan revealed bilateral pulmonary embolism. She received thrombolysis with alteplase and was started on rivaroxaban. Five days after discharge, however, she was readmitted with severe vaginal bleeding.

**Discussion and conclusion:**

We describe a case of submassive pulmonary embolism, treated with thrombolysis and anticoagulation, who developed severe vaginal bleeding after stopping the contraceptive pill. This case highlights the importance of detailed menstrual history taking when initiating anticoagulation in women. Discontinuation of oral contraceptives, while important in reducing the risk of recurrent thrombosis, could be postponed until the end of the recommended course of anticoagulation and until a safe alternative form of contraception has been established, if required.

## Background

Pulmonary embolism is a common and potentially fatal condition. Pulmonary embolism usually arises from a deep venous thrombosis of the lower extremities. Rarely it originates from the pelvic, renal, upper extremity veins or right heart. The risk factors for venous thromboembolism (VTE) are summarized in Virchow’s triad: hypercoagulability, stasis, and endothelial injury. Exogenous estrogens, either as contraceptives or as post-menopausal hormone replacement, induce a prothrombotic state and are thus associated with an increased risk of VTE [1].

## Case presentation

A 47-year-old woman, non-smoker, with history of asthma and pollen allergy contacted the emergency services for subacute dyspnea, exercise intolerance and chest tightness. Her daily medication consisted of: desloratadine, a beclomethason/formoterol inhaler and an ethinylestradiol/levonorgestrel contraceptive, which she took on a continuous basis (without pill-free days) because of pre-menopausal menometrorrhagia. She had been feeling unwell for two days and thought her symptoms were due to an asthma attack. She had already tried increasing her inhaler, but without effect. Two days earlier she had returned from holiday after an 18-hour bus ride. She had taken the same bus on the outward journey 10 days earlier.

**Fig. 1:**
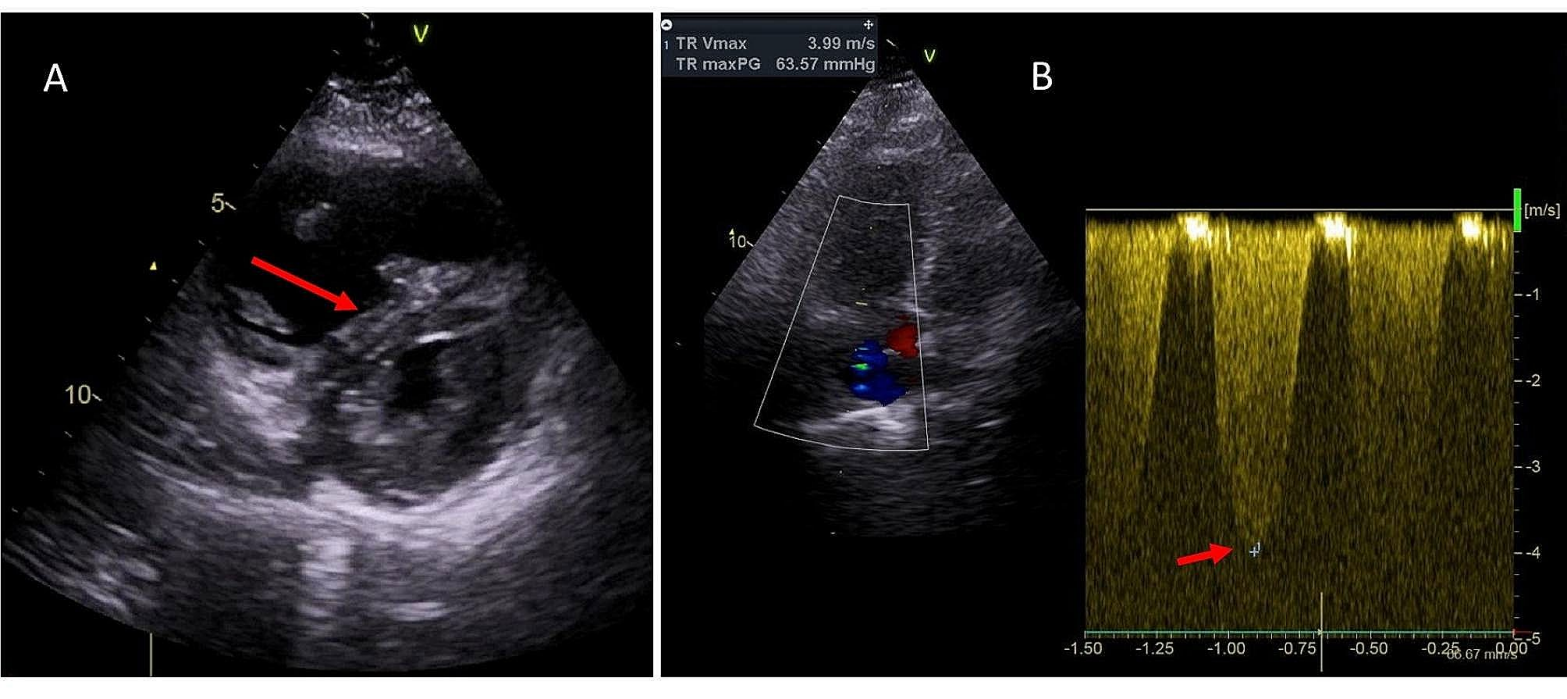
Transthoracic echocardiogram: (**A**) Parasternal short axis image, showing dilated right ventricle with systolic D-shaping of the interventricular septum (arrow); (**B**) Continuous wave doppler in apical four chamber view, showing estimated pulmonary artery systolic pressure of 64 mmHg + central venous pressure, as measured by tricuspid regurgitation jet velocity (arrow)

On arrival, the first responders team found her sitting on the ground in respiratory distress, tachypneic, tachycardic, hypoxic, hypotensive, and afebrile. Her vital signs were: respiratory rate 36 breaths per minute, heart rate 142 beats per minute (bpm), oxygen saturation 72% on room air, blood pressure 64/43 mmHg and temperature 35.8 °C. ECG showed sinus tachycardia, without Q waves or ischemic ST/T changes. The patient was given 12 L/m of oxygen via face mask and 500 mL of normal saline and was brought to the hospital. Repeat ECG showed similar findings, lab (results of which were only available later) showed hemoglobin of 14.0 g/dL, troponin 330 ng/L, d-dimers 7509 mcg/L, C-reactive protein 30.4 mg/L and creatinine of 1.31 mg/dL, corresponding to estimated glomerular filtration rate of 48 mL/min/1.73m^2^. Quick-look echocardiogram showed a nondilated and normocontractile left ventricle, a dilated right ventricle with leftward shift of the interventricular septum and pulmonary hypertension with an estimated right ventricular systolic pressure of 64 mmHg + central venous pressure (Fig. 1). Inferior caval vein was plethoric without respiratory variation. There was no severe valvular pathology. At that moment, blood pressure was 142/95 mmHg, heart rate 139 bpm and oxygen saturation 97% while breathing 12 L/m oxygen via face mask.

**Fig. 2:**
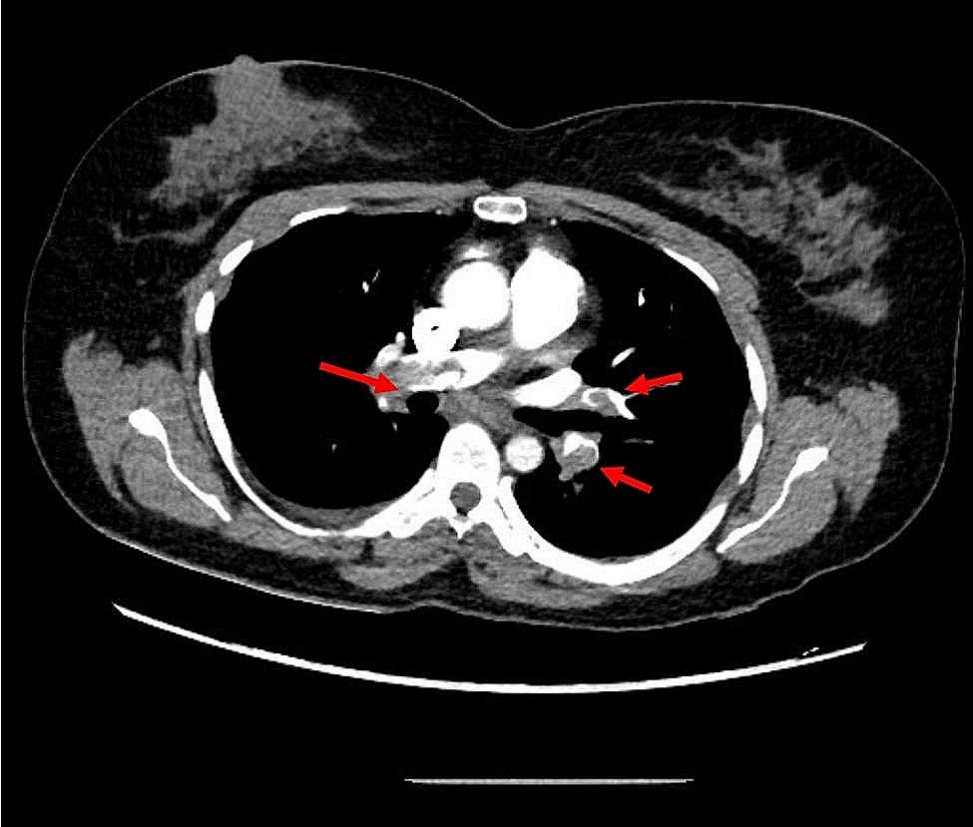
Computerized tomography scan with intravenous contrast, showing pulmonary embolism (arrows) in right and left pulmonary artery

Acute pulmonary embolism was suspected. The patient was given 80 mg of enoxaparin (weight = 83 kg) and an urgent computerized tomography scan with intravenous contrast was performed, which confirmed the diagnosis of bilateral pulmonary embolism (Fig. 2**).**

The Pulmonary Embolism Severity Index (PESI) score, calculated with the help of an online tool [2], was 167: very high risk. Based on hemodynamic compromise, right ventricular dysfunction on echocardiogram and a very high-risk PESI score, thrombolysis was administered. Alteplase was given as a 10 mg bolus and 90 mg infusion over 2 hours and the patient was admitted to the cardiac intensive care unit. Her condition gradually improved over the next few hours, with a decrease in heart rate from 130 bpm to 80 bpm and normalization of serum creatinine to 0.81 mg/dL (corresponding to eGFR of 85 mL/min/1.73 m^2^) by the second day. Repeat echocardiography showed a marked decrease in right ventricular dimensions and lowering of estimated right ventricular pressure to 27 mmHg + central venous pressure. In addition to the pulmonary embolism, the patient was found to have extensive deep venous thrombosis of the right femoral vein. Thrombophilia screening revealed a Factor V Leiden mutation. After two days of enoxaparin, 80 mg (1 mg/kg) twice daily, she was switched to rivaroxaban, 15 mg twice daily and at day 6 she was discharged with this therapy for a total of 21 days, after which she should decrease the dose to 20 mg once daily. She was instructed to discontinue her combined oral contraceptive pill indefinitely.

Five days later, however, the patient was re-admitted to the hospital with pre-syncope. She was looking pale. During the few days between hospital admissions, she had had severe vaginal bleeding, with need for hygienic pad change every few hours. Vital signs were: blood pressure 102/74 mmHg, heart rate 114 beats per minute, respiratory rate 18 per minute, saturation 99% on room air, temperature 36.7 °C. There was no hematuria, melaena or hematochezia. Hemoglobin was 6.3 g/dL, ß-HCG negative, creatinine 0.96 mg/dL and C-reactive protein 2.1 mg/L. INR was 1.4 (12 h after last intake of rivaroxaban).

**Fig. 3:**
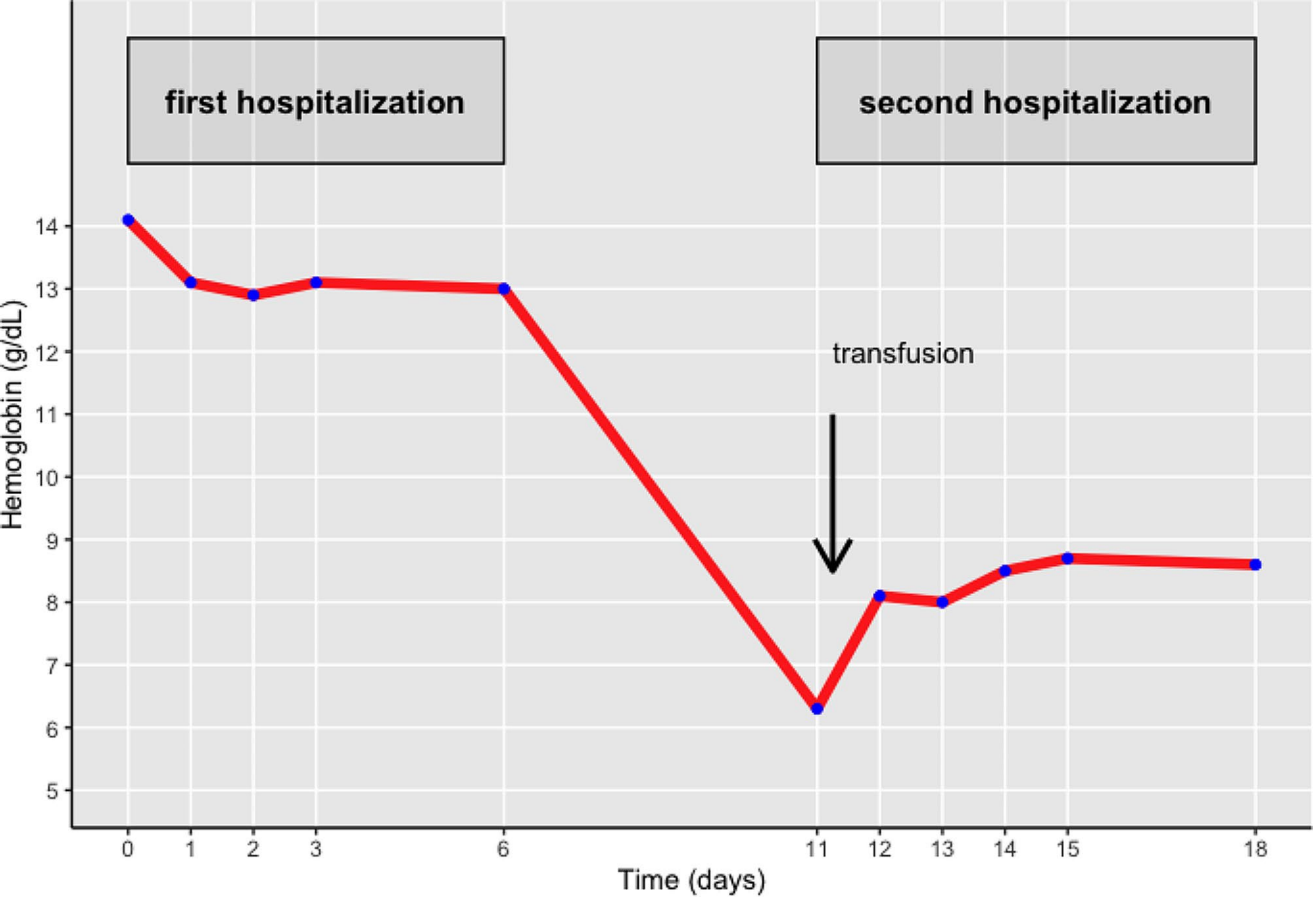
Graph showing evolution of Hemoglobin (y-axis, in g/dL) over Time (x-axis, in days). First hospitalization (admission on day 0, discharge on day 6) and second hospitalization (admission on day 11 and discharge on day 18) are indicated. Vertical arrow indicates transfusion of 2 units packed cells and 500 mL of normal saline

Figure 3 shows the evolution of anemia since the previous hospitalization. 500 mL of normal saline and two units of packed cells were infused. Rivaroxaban was withheld for 5 days and substituted for prophylactic dose enoxaparin, 40 mg once daily. Lynestrenol, an oral progestin, was administered at 5 mg twice daily for two weeks, and then switched to nomegestrol 5 mg once daily, to be taken continuously. An oral iron supplement was started to replete the iron stores. The patient was discharged on day 7.

## Discussion and conclusion

Heavy menstrual bleeding is a common complication of anticoagulation. Detailed menstrual history taking is required prior to initiating anticoagulation in women, as it can identify women at higher risk for this complication. Treatment consists of judicious reevaluation of the need for and kind of anticoagulation, transfusion in case of severe anemia, iron supplementation and hormonal therapy.

Interruption of anticoagulation is not ideal in the context of an acute VTE. However, choice and dose of anticoagulation should be reevaluated. Rivaroxaban is associated with a higher risk of heavy menstrual bleeding than other anticoagulants; a switch should be considered [3]. Patients should be evaluated for iron deficiency and treated.

The timing of discontinuation of oral contraceptives poses a dilemma. On the one hand, estrogen-containing oral contraceptives are clearly associated with an increased risk of VTE. On the other hand, discontinuation carries a risk of pregnancy, withdrawal bleeding and heavy menstrual bleeding at the next cycle. Moreover, continuation of a combined oral contraceptive pill in women with VTE appears to be safe, as long as these women are taking anticoagulation. A subgroup analysis of women under 60 years treated with anticoagulants for acute VTE included in the EINSTEIN DVT and PE trials, showed a similar rate of recurrent VTE in those who did and did not receive hormonal therapy [4]. Therefore, the International Society on Thrombosis and Haemostasis (ISTH) recommends continuation of oral contraception for the duration of the anticoagulant therapy [5].

In conclusion, we described a typical case of submassive pulmonary embolism, treated with thrombolysis and anticoagulation, in the context of immobilization and combined oral contraceptive use in a woman with Factor V Leiden, who developed severe vaginal bleeding after stopping the contraceptive pill. This case highlights the importance of taking a menstrual history before initiating anticoagulation in women. Discontinuation of oral contraceptives, while important in reducing the risk of recurrent thrombosis, could be postponed until the end of the recommended course of anticoagulation and until a safe alternative form of contraception has been established, if required.

## Data Availability

Data sharing is not applicable to this article as no datasets were generated or analyzed for this case report.

## Abbreviations

ß-HCG: ß- human chorionic gonadotrophin
ECG: electrocardiogram
eGFR: estimated glomerular filtration rate
INR: international normalized ratio
PESI: Pulmonary Embolism Severity Index
